# Maternal and clinical predictors of insulin requirement in gestational diabetes mellitus (GDM): A cross-sectional study

**DOI:** 10.6026/973206300213628

**Published:** 2025-10-31

**Authors:** Anbumathi Suriyamoorthy, Ciba Vaitheeshwari Baskaran, Nadhu Krishna Maheswaran Nair, Robin S, Maheshlakhiru Murugaiyan, Charru Pooja Srinivasan, Dilip S, Hema Loshani Suresh, Pushpa Saravanan, Dharmarajan Panneerselvam

**Affiliations:** 1 Department of Institute of Diabetology, Madras Medical College, Chennai, India; 2 Department of Medicine, Medway Hospitals, Chennai, India; 3 Department of Medical Gasteroenterology, VGM Hospitals, Coimbatore, India

**Keywords:** Gestational diabetes mellitus, insulin therapy, risk factors, maternal outcomes, lifestyle modification

## Abstract

Gestational diabetes mellitus (GDM) poses a significant challenge due to its variable insulin requirements and impact on pregnancy outcomes. Therefore, it is of interest to identify maternal, clinical, and lifestyle factors associated with insulin use among 386 women diagnosed with GDM. Detailed data on demographics, anthropometry, family history, comorbidities, diet, physical activity, and obstetric outcomes were analyzed. Higher pre-pregnancy weight, family history of diabetes, insulin resistance markers, and co-morbid conditions were significantly linked to insulin requirement. Early identification of high-risk women can enable timely interventions and improve maternal and fetal outcomes.

## Background:

Gestational diabetes mellitus (GDM) is a carbohydrate intolerance of variable severity that develops or is first recognized during pregnancy, posing significant health risks for both mother and fetus [1]. The rising prevalence of GDM globally, particularly in South Asian populations, is linked to increasing maternal age, urban lifestyles and obesity [2]. GDM is associated with a higher risk of complications such as macrosomia, hypertensive disorders and cesarean delivery, while also serving as a precursor for type 2 diabetes in both mother and child [3]. Management of GDM typically begins with lifestyle modifications including diet and exercise; however, a subset of women requires pharmacologic interventions such as insulin or metformin for optimal glycemic control [4]. Identifying which patients are more likely to need insulin therapy remains a clinical challenge, especially in low-resource settings where routine monitoring may be limited [5]. Several maternal factors, including pre-pregnancy weight, family history of diabetes, co-existing comorbidities and insulin resistance, may influence treatment needs [6]. Early recognition of these risk indicators can aid in timely initiation of therapy and reduce adverse outcomes [7]. Furthermore, understanding lifestyle and dietary patterns unique to regional populations can refine individualized care strategies [8]. This study was undertaken to evaluate the maternal, clinical and lifestyle determinants of insulin requirement in women with GDM and to explore their association with maternal and fetal outcomes. Therefore, it is of interest to study the maternal, clinical and lifestyle predictors of insulin requirement in women with GDM to guide timely interventions and improve outcomes.

## Materials and Methods:

This cross-sectional observational study was conducted among 386 pregnant women diagnosed with gestational diabetes mellitus (GDM) attending antenatal clinics at a tertiary care institution. Data were collected from clinical records, structured patient interviews and follow-up documentation. Diagnosis of GDM was based on OGCT/OGTT results interpreted as per standard clinical guidelines. Detailed information was gathered on age, obstetric history and family history of diabetes, pre-pregnancy weight, gestational weight gain, body mass index and co-morbidities. Management modalities were documented, including meal plans, metformin use, or insulin therapy, based on glycemic status and clinician discretion. Dietary habits, physical activity and presence of clinical markers of insulin resistance (such as acanthosis nigricans or skin tags) were also noted. Obstetric scan reports were reviewed for fetal growth or anatomical abnormalities and outcomes were categorized as vaginal delivery or lower segment cesarean section (LSCS). Descriptive and analytical statistics were used to examine associations between clinical and lifestyle variables with insulin requirement and delivery outcomes. All analyses were performed using cleaned and anonymized data, ensuring patient confidentiality throughout.

## Results:

A total of 386 women diagnosed with gestational diabetes mellitus (GDM) were included, with a mean age of 28.9 years. Most participants were multiparous and conceived spontaneously and over a quarter required insulin therapies during pregnancy. Family history of diabetes was more prevalent in women aged 26-35 years and universally present in those above 40 years. Higher pre-pregnancy weight was associated with greater insulin use, whereas women with lower weight were mostly managed without pharmacologic therapy. Engagement in physical activity varied by treatment modality, with the highest exercise participation observed among women on metformin and minimal activity among those managed by diet alone. Total gestational weight gain influenced delivery outcomes, with excessive weight gain (>15 kg) correlating with higher LSCS rates, while moderate gain favored vaginal delivery. Abnormal ultrasound findings were linked to an increased likelihood of surgical delivery. The presence of co-morbidities, clinical markers of insulin resistance and a positive family history were associated with slightly higher insulin requirements. Diet type also influenced insulin use, with vegetarians showing marginally higher rates than non-vegetarians. Collectively, these findings highlight that maternal characteristics, lifestyle factors and clinical markers contribute to treatment needs and delivery outcomes in GDM.

Table 1 (see PDF) demonstrates the distribution of family history (FH) of diabetes across different age groups. Among women aged ≤25 years, 22% had a positive family history, while in the 26-30 and 31-35 age groups, this increased to 29.3% and 24.0%, respectively. Notably, all women aged above 40 had a positive FH. Table 2 (see PDF) outlines the association between pre-pregnancy weight and the type of treatment received during pregnancy. Women with higher pre-pregnancy weight (>70 kg) had the highest proportion of insulin use (20.5%). In contrast, the majority of women in the ≤50 kg and 51-60 kg categories were managed without pharmacologic therapy. Table 3 (see PDF) explores the relationship between treatment modality and engagement in physical activity during pregnancy. Nearly half of those on insulin reported regular exercise, while the highest proportion of exercise was noted among those on metformin (66.7%). The majority of women managed through dietary measures alone (meal plan) reported no exercise during pregnancy. Table 4 (see PDF) presents the relationship between total gestational weight gain and the mode of delivery. Among women who gained more than 15 kg, the rate of LSCS was highest at 26.7%, with no recorded vaginal deliveries in that group. Conversely, those who gained between 6-10 kg had a higher proportion of NVDs (17.6%) compared to LSCS (10.2%), suggesting moderate weight gain might be more favorable for vaginal delivery. Table 5 (see PDF) shows the association between ultrasound scan findings and delivery type. Among those with abnormal scans, 41.7% underwent LSCS and 33.3% delivered vaginally, whereas those with normal scans had a much lower LSCS rate (12.0%). This suggests a notable correlation between sonographic abnormalities and surgical delivery. Table 6 (see PDF) highlights the relationship between the presence of co-morbid conditions and the use of insulin in GDM management. Women with co-morbidities had a slightly higher rate of insulin use (15.2%) compared to those without (11.7%). Table 7 (see PDF) presents the association between the method of conception and insulin use. All women who conceived via assisted reproductive techniques (e.g., IVF/IUI) were managed without insulin. Among those who conceived spontaneously, 13.0% required insulin therapy, while the rate was slightly higher (16.3%) among those with unknown conception details. Table 8 (see PDF) examines the relationship between dietary patterns and insulin use. Among non-vegetarian participants, 13.6% required insulin, while insulin use was slightly higher among vegetarians (20.0%). The lowest rate of insulin therapy (4.0%) was observed in those with unclassified diet data. Table 9 (see PDF) highlights the association between visible markers of insulin resistance (IR) and insulin therapy. Women with clinical markers of IR (such as acanthosis nigricans or skin tags) had a higher likelihood of requiring insulin (16.0%) compared to those without such markers (11.2%). Table 10 (see PDF) summarizes the association between family history of diabetes and insulin use. Among women with a positive family history, 18.6% required insulin, in contrast to 11.4% of those without a familial diabetic background. This indicates a trend toward increased insulin requirement in genetically predisposed individuals.

## Discussion:

The present study highlights key maternal and clinical determinants associated with insulin requirement among women diagnosed with GDM [9]. Higher pre-pregnancy weight and greater gestational weight gain were both significantly linked with the need for insulin therapy, consistent with existing literature emphasizing the metabolic burden of adiposity on glucose regulation [10]. Family history of diabetes and clinical markers of insulin resistance also emerged as strong predictors, suggesting a genetic and phenotypic predisposition to more severe glycemic dysregulation [11]. While lifestyle factors such as diet and exercise showed moderate influence, their variability and self-reported nature may have affected the strength of observed associations [12]. Interestingly, women with abnormal obstetric scans and excessive weight gain were more likely to deliver by cesarean section, underscoring the downstream impact of poor glycemic control [13]. Assisted reproductive techniques did not show a higher insulin requirement, indicating that conception type may not independently predict severity of GDM [14]. These findings reinforce the need for early risk stratification in GDM patients to guide timely therapeutic decisions Tailoring management strategies based on predictive indicators could improve both maternal and neonatal outcomes in resource-limited settings.

## Conclusion:

Maternal factors such as pre-pregnancy weight, family history of diabetes, insulin resistance markers and co-morbidities are significant predictor of insulin requirement in gestational diabetes mellitus is shown. Early identification and proactive management of high-risk women can help prevent adverse obstetric outcomes and improve overall pregnancy care in GDM.
